# Supplementary material for: Time series analyses with psychometric data
Source: PLoS One. 2020 Apr 16;15(4):e0231785. doi: 10.1371/journal.pone.0231785 (PMC7162519; doi:10.1371/journal.pone.0231785)
Supplement: Attachment — Submitted filename: Response to Reviewers.doc [file pone.0231785.s006.doc]

# Response to Reviewers

| Reviewer 1 | 1 | “…*only one* dataset for the conducted experiments; …experiments with at least *two additional (and diverse)* datasets are expected to support the claims of the authors”. | 241-249: Explanations that  dozens of different data types were generated in the Monte Carlos simulations of the present study. |
| --- | --- | --- | --- |
|  | 2 | Typos | corrected |
|  | 3 | “Contribution of the study is unclear…” | explained: 309-360 |
| Reviewer 2 | 1 | Equation 2 | improved |
|  | 2 | “…provide two-three real-life applications of the proposed study…” | provided: 309-360 |

| Editor | 1 | PLOS ONE's style requirements | checked |
| --- | --- | --- | --- |
|  | 2 | A study's minimal data set | provided as supplementary files S2_File.R, S3_File.R, S4_File.R |
|  | 3 | Uploading the figure files to the PACE | done |
