# Supplementary material for: Time series analyses with psychometric data
Source: PLoS One. 2020 Apr 16;15(4):e0231785. doi: 10.1371/journal.pone.0231785 (PMC7162519; doi:10.1371/journal.pone.0231785)
Supplement: Attachment — Submitted filename: Response to Reviewers.doc [file pone.0231785.s007.doc]

# Response to Reviewers

| Reviewer 1 | 1 | “… authors should perform additional experiments using *real* data to demonstrate their point. Using simulations to generate data is not enough, since in most domains, the actual distribution differs from the one used in simulations. Further evidence should be provided if this is not the case for psychometric data.” | - 197-232: Explanations that the aim of the study can be achieved only with simulated data. - The data generated in the study are typical for psychology: non-metric, with missing values... The evaluation method used here is a standard statistical procedure to answer the questions of the study adequately. - 65-68: Please note that numerous empirical examples with psychometric data are already provided elsewhere. |
| --- | --- | --- | --- |
| Reviewer 3 | 1 | “ ..the data sets for S2 to S4 are really not data sets but simulated examples.” | The data sets in S2 to S4 provide all simulated data of the study. For instance, in S2 the commands “X=ordinal_10_m(ls$X),Y=ordinal_10_m(ls$Y)” at the end of the code generate 10 cointegrated ordinal measured bivariate systems with length 100 and β_0_=-1, because the set parameters are N=10, T=100, b1=1, b2=1. Changing the parameters to, for example, N=1000 and T=500 generates 1000 cointegrated systems with length 500. With “X1_t=X[,1], Y1_t=Y[,1], CI_1=cbind(X1_t, Y1_t)“ one just gets the first system or with “X1_t=X[,10], Y1_t=Y[,10], CI_1=cbind(X1_t, Y1_t)“ the 10^th^ one. I included the commands print(X) and print(Y) in the codes to demonstrate this (the simulated data can be viewed). Employing a for-loop one can access all N systems successively.  Please note that the simulated examples in Fig 1 (S1) are metric, S2 to S4 provide simulations codes for generating non-metric (interval, ordinal…) data of the study. |
|  | 2 | “The real life types of examples to which these procedures could be applied are descriptively outlined by the authors on lines 309 to 360. It would have been more helpful to have actual data associated with these types of examples to demonstrate the procedures numerically.” | The data of the empirical example in Fig 1 are provided as S5_File.csv (S5). The R codes for analyzing the data are included in the appendix (S1: S1_File.R) to show that the Johansen test handles simulated and non-simulated data equally. There are no fundamental differences between these data and other time series from studies outlined on lines 309 to 360.  Please note that numerous empirical examples with psychometric data are already provided elsewhere (see 65-68) and the aim of the study can be achieved only with simulated data (197-232). |
